# Vitamin K_2_ in Electron Transport System: Are Enzymes Involved in Vitamin K_2_ Biosynthesis Promising Drug Targets?

**DOI:** 10.3390/molecules15031531

**Published:** 2010-03-10

**Authors:** Michio Kurosu, Eeshwaraiah Begari

**Affiliations:** Department of Microbiology, Immunology, and Pathology, College of Veterinary Medicine and Biomedical Sciences, Colorado State University, 1682 Campus Delivery, Fort Collins, CO 80523-1682, USA; E-Mail: begari.eeshwaraiah@colostate.edu (E.B.)

**Keywords:** vitamin K_2_, menaquinone, electron transport system, menaquinone biosynthesis, MenA, menaquinone biosynthesis inhibitor, electron transport system inhibitor, antibacterial agent, multidrug-resistant bacteria

## Abstract

Aerobic and anaerobic respiratory systems allow cells to transport the electrons to terminal electron acceptors. The quinone (ubiquinone or menaquinone) pool is central to the electron transport chain. In the majority of Gram-positive bacteria, vitamin K_2_ (menaquinone) is the sole quinone in the electron transport chain, and thus, the bacterial enzymes catalyzing the synthesis of menaquinone are potential targets for the development of novel antibacterial drugs. This manuscript reviews the role of vitamin K in bacteria and humans, and especially emphasizes on recent aspects of menaquinones in bacterial electron transport chain and on discoveries of inhibitor molecules targeting bacterial electron transport systems for new antibacterial agents.

## 1. Introduction

Vitamin K is a lipid-soluble essential vitamin that is stable to air but susceptible to air under sunlight. The "K" is derived from the German word “koagulation”. Coagulation refers to the process of blood clot formation. Henrick Dam and Edward Doisy were co-awarded the Nobel Prize in 1943 for their discovery of vitamin K (the "koagulations" vitamin) and the elucidation of its structure. Natural forms of vitamin K, vitamin K_1_ (phylloquinone) and vitamin K_2_ (menaquinone), exist in the human liver (approximately 10% of phylloquinone in the total vitamin K) and other tissues at very low concentrations; vitamin K_1_ concentrates in the liver while vitamin K_2_ is well distributed to other tissues [1]. Phylloquinone is derived from dietary intake and menaquinones are produced by intestinal bacteria. There is no direct evidence for the utility of menaquinones by humans, however, it is believed that menaquinones are utilized for the synthesis of blood-clotting factors when phylloquinone is depleted [2]. In addition, menaquinones are known to be more effective than phylloquinone with respect to osteroclastogenesis, hypocholesterolemic effects, and ability to slow atherosclerotic progression. 

Menaquinones play important roles in electron transport, oxidative phosphorylation, active transport, and endospore formation in bacteria. In addition to these functions, the variations in the inherent structures of menaquinones and their uneven distributions among bacteria are considered important in bacterial taxonomy [3]. Recently, menaquinone biosynthesis has received considerable attentions as drug targets for multidrug-resistant Gram-positive pathogens including *Mycobacterium tuberculosis*. 

## 2. General Structures of Vitamin K

**Figure 1:**
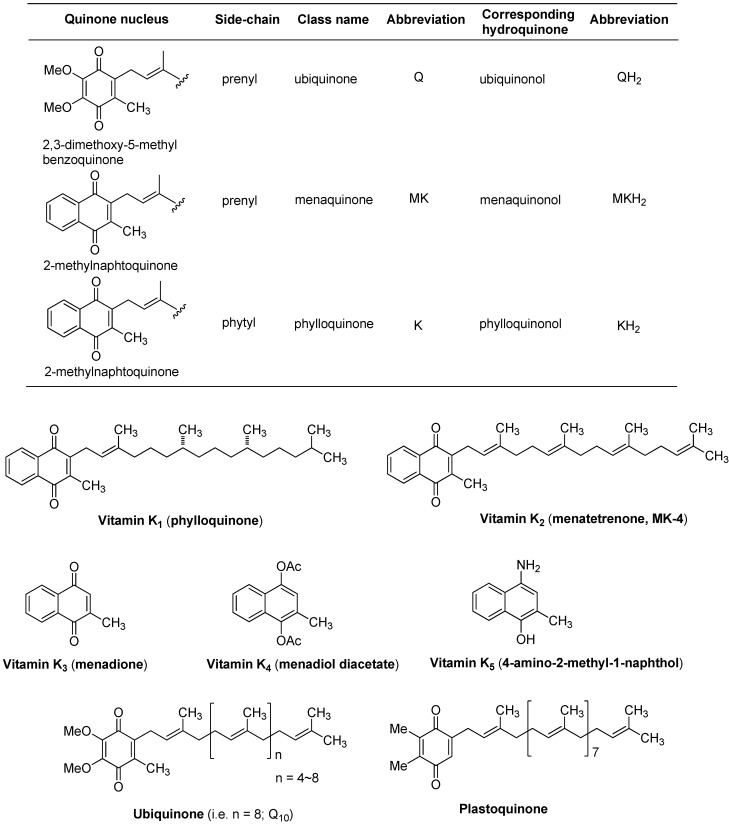
Names for quinones and structures of naturally occurring benzoquinones (ubiquinone and plastoquinone) and naphthoquinones (vitamin K_1_ and K_2_) and representative synthetic vitamin Ks (vitamin K_3_, K_4_, and K_5_).

Although the name vitamin K is not a designated chemical name according to IUPAC practice, and because bicyclic form of quinones cannot easily be designated, the names of vitamin K_1_ and vitamin K_2_ are replaced by appropriate chemical names such as phylloquinone and menaquinone, respectively [4]. 2-Methyl-1,4-naphthoquinone is called vitamin K_3_ or menadionone. 2,3-Dimethoxy-5-methylbenzoquinone is called ubiquinone (ubiquinone, originally a denotation for the “ubiquitous quinine”). The 2,3-dimethylbenzoquinone nucleus is observed in plastoquinone which is involved in the electron transport chain in photosynthesis. Their abbreviations of ubiquinone, menaquinone, and phylloquinone are Q, MK, and K, respectively (Figure 1). Different nomenclatures of quinones and naphthoquinones have been used in different scientific fields. 

Plants and some cyanobacteria synthesize phylloquinone. Bacteria synthesize a range of vitamin K forms (but not vitamin K_1_) using repeating isoprene (5-carbon) units in the side chain of the molecule (*vide infra*). These forms of vitamin K are designated menaquinone-n (MK-n), where n stands for the number of 5-carbon units in the structure. Menaquinones (MK-n) are collectively referred to as vitamin K_2_. Vitamin K_1_ and K_2_ have the common structure of a 2-methyl-1,4-naphthoquinone system and are structurally different in the number of isoprene units in the side chain and in their degree of unsaturation (Figure 1). Menaquinones with side chains of up to 15 isoprene units have been reported in literatures. For example, MK-8 is predominant in *Escherichia coli* but *Mycobacterium tuberculosis* utilizes MK-9 as a lipid-soluble electron carrier. Menaquinones possessing 2 to 13 isoprene units have been found in human and animal tissues. Several synthetic vitamin Ks are commercially available, and representative synthetic vitamin Ks such as vitamins K_3_, K_4_, and K_5_ are used in many areas including the pet food industry (vitamin K_3_) and to inhibit fungal growth (vitamin K_5_). 

## 3. Vitamin K1 in Humans

The role of vitamin K as a cofactor in blood coagulation stems from the post-translational modification of a number of plasma proteins such as factors II (prothrombin), VII, IX, X, proteins C and S, as well as Gla (γ-carboxyglutamic acid) proteins has been well-documented. 

**Figure 2:**
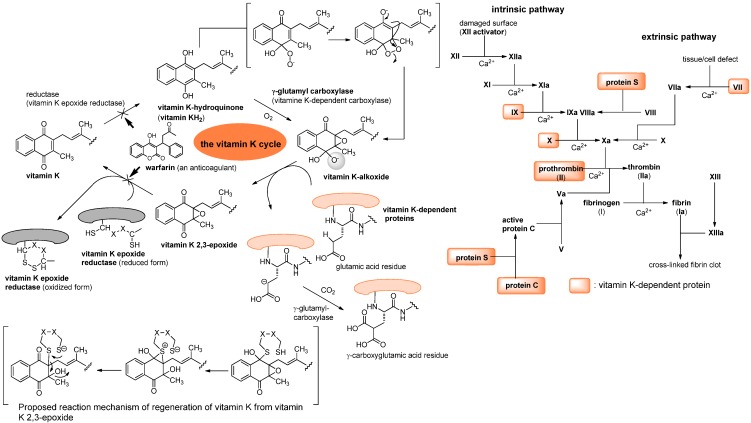
Vitamin K as a cofactor in blood coagulation systems.

Blood coagulation is regarded as a series of consecutive proenzyme (inactivate enzyme precursor) to enzyme conversion, often called a cascade reaction. The reduced form of vitamin K (hydroquinone form), acts as a cofactor in the enzymatic carboxylation by γ-glutamyl carboxylase, forming γ-carboxyglutamic acid in plasma proteins [5,6,7]. In the process of carboxylation, vitamin K 2,3-epoxide is formed, which is reduced back to vitamin K by the cysteine residues of vitamin K epoxide reductases. The role of vitamin K-dependent γ-glutamyl carboxylase in the blood coagulation cascade is summarized in Figure 2. The vitamin K-dependent γ-glutamyl carboxylase catalyzes the processive carboxylation of specific glutamate residue(s) in a number of proteins related to blood coagulation and bone. To date, several γ-carboxyglutamic acid containing proteins including the blood coagulation factors have been characterized. In all cases, the precise function of their γ-carboxyglutamic acid residue is not known, however, the presence of the γ-carboxyglutamic acid residues in their proteins is essential for functional activity. Extracellular calcium ion plays an important role in the blood-clotting cascade and is necessary for the formation of fibrin from fibrinogen; the conversion of prothrombin to thrombin, and as a cofactor for factors V, VII, VIII, IX, X, and XIII [8]. All the vitamin K-dependent proteins bind Ca^2+^. Thus, γ-carboxyglutamic acid residues of vitamin K-dependent proteins are considered to serve as effective divalent counter ions to bind Ca^2+^. However, a deficiency of vitamin K is rare due to the fact that 1) bacteria synthesize within the human body, and 2) vitamin K is continually recycled in our cells (see, vitamin K cycle in Figure 2). 

The drug warfarin is a vitamin K antagonist that inhibits vitamin K epoxide reductase, interfering with the reduction of the epoxide and halting the cycling back to the hydroquinone intermediate, thereby interrupting the activation of blood coagulation factors [9,10,11]. Other vitamin K-dependent proteins not involved in blood-clotting, such as osteocalcin (play a role in mineralization and calcium ion homeostasis), or matrix Gla protein (calcium-binding proteins that participate in the organization of bone tissue), may also be affected by warfarin.

In addition to the pivotal role of vitamin K in the blood-clotting cascade and vitamin K-dependent proteins (*vide supra*), the potential role of vitamin K in the increase of bone mass [12,13], antioxidant properties of vitamin K [14], the vitamin K deficiency and biosynthesis of cholesterol and steroid hormones [15], and relationship between vitamin K and liver or prostate cancer [16,17,18] have been studied. It is known that vitamin K is not only distributed in the liver and bones but also abundantly distributed in the brain, kidney, and gonadal tissues [19,20]. However, the role of vitamin K in these tissues is not well understood. In addition, distribution of vitamin K varies depending on the structure of the side chain moiety. In humans, vitamin K_1_ is distributed to all tissues with relatively high levels in the liver, heart and pancreas (medians, 10.6 (4.8), 9.3 (4.2), 28.4 (12.8) pmol(ng)/g wet weight tissue); low levels (<2 pmol/g) were found in the brain, kidneys and lungs. Menaquinone-4 (MK-4) is also distributed to most of the tissues; its levels exceed the vitamin K_1_ levels in the brain and kidneys (median, 2.8 ng/g) and are equal to that in pancreas. The liver, heart and lung are low in MK-4. MK-6~11 are found in the liver. Trace amounts of MK-6~9 are found in the heart and pancreas. Total vitamin K in human plasma was reported to be 0.47~1.19 nmol/L [1,21,22]. 

## 4. Ubiquinone in Humans

**Figure 3:**
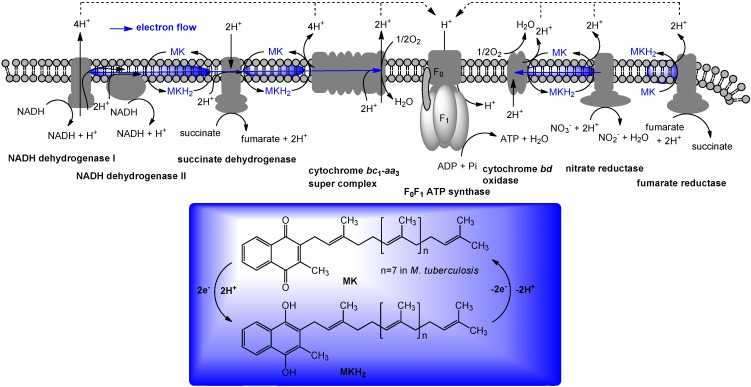
The electron flow system from *M. tuberculosis* as example of menaquinone mediated energetic pathway.

The most common ubiquinone in human mitochondria is Q_10_ [or Coenzyme Q_10_ (CoQ_10_), the 10 refers to the number of isoprene units, Figure 1). Different organisms biosynthesize ubiquinone possessing different length of the side chain. Humans and some *Schizosaccharomyces* spp. produce Q_10_ (only 2~7% of Q_9_ is present in human tissues), mouse biosynthesizes Q_9_, *Escherichia coli* biosynthesizes Q_8_, and *Saccharomyces cerevisiae* produces Q_6_. In eukaryotes ubiquinones are found in the inner mitochondrial membrane and in other membranes such as the endoplasmic reticulum, Golgi vesicles, lysosomes and peroxisomes. Although Q_10_ is found in virtually every cell in the human body where energy is produced, highest concentration is found in the heart and the liver because they contain the most mitochondria per cell. Coenzyme Q_10_ is an essential vitamin-like substance (Q_10_ can be synthesized in human body), and its primary function is to generate ATP in the mitochondria. Coenzyme Q_10_ is the coenzyme for at least three mitochondrial enzymes (complexes I, II and III) in the electron transport system (see a schematic bacterial electron transport system in Figure 3). Function of ubiquinone as a component of mitochondrial respiratory chain is well established. Peter Mitchell received the Nobel Prize in 1978 for his contribution to the understanding of biological energy transfer through the formulation of “the chemiosmotic theory”, which includes the vital protonmotive role of Q_10_ in energy transfer systems [23,24,25]. In addition to the functions of Q_10_ as an electron carrier in the respiratory chain and as an antioxidant (free radical scavenger to reduce oxidative damage to tissues as well as significantly inhibit the oxidation of LDL cholesterol) [26,27], Q_10_ has been reported to regulate global gene expression in skeletal muscle and the functions of Q_10_ in relation to aging process have been discussed [28,29]. Decreased levels of Q_10_ have been noted in a wide variety of diseases in both animal and human studies. Coenzyme Q_10_ supplementation is very effective for those who are taking lipid-lowering drugs such as HMG-CoA reductase inhibitors (*i.e*., statins) or bold glucose-lowering drugs (*i.e*., tolazamide and glyburide), because these drugs inhibit the production of coenzyme Q_10_ in the liver. Although some study showed that dominant source of Q_10_ in humans is biosynthesis [30], the relative contribution of Q_10_ versus dietary Q_10_ is under investigation. In ubiquinone synthesis 4-hydroxybenzoic acid (derived from tyrosine or phenylalanine), is condensed with polyprenyl diphosphate which is catalyzed by *para*-hydroxybenzoate (PHB) polyprenyltransferase encoded by the *COQ_2_* gene. Most of the genes encoding prenyltransferases which synthesize polyisoprenoid chains, have been cloned. The prenylated 4-hydroxybenzoic acid undergoes functional modifications such as hydroxylations, *O*-methylations, methylation, and decarboxylation. Although not all the genes that encoded enzymes in ubiquinone biosynthesis in higher eukaryotic cells have been cloned, it was reported that they are similar to the yeast genes [31]. 

## 5. The Role of Vitamin K_2_ in Electron Transport

In prokaryotes, especially in Gram-positive bacteria, menaquinone will transfer two electrons in a process of aerobic or anaerobic respiration. Respiration occurs in the cell membrane of prokaryotic cells. Electron donors, with the help of an enzyme, will transfer two electrons to menaquinone. Menaquinone, with the help of another enzyme, will in turn transfer these two electrons to an electron acceptor. Schematic electron flow mediated by menaquinone in *M. tuberculosis* is illustrated in Figure 3. The exact organization of enzymes in respiratory chains will vary among different bacteria; this illustration is intended to demonstrate the basic features of the process. Although NADH is the most important electron donor in eukaryotes, bacteria can use a number of different electron donors, a number of different dehydrogenases, a number of different oxidases and reductases, and a number of different electron acceptors. As illustrated in Figure 3, electrons are transported along the membrane through menaquinone and a series of protein carriers (*i.e*., cytochrome *bc_1_* complex). Concomitantly, protons are translocated across the cell membrane, from the cytoplasm to the periplasmic space. The cell membrane is impermeable to protons, except through ATP synthases. When protons move through these complexes, energy releases by their passage in coupled to synthesis of ATP from ADP and phosphate. Thus, the lipid-soluble electron carriers (lipoquinones) occupy a central and essential role in electron transport, and thus, ATP synthesis [32,33]. The lipoquinones involved in the respiratory chains of bacteria consist of menaquinones and ubiquinones. From the taxonomic studies it is evident that the majority of Gram-positive bacteria including *Mycobacterium* spp. utilize only menaquinone in their electron transport systems [34], and menaquinone biosynthesis is essential for survival of Gram-positive bacteria [3,35,36]. Menaquinone synthesis has been extensively studied in *E. coli* (due in part to the availability of the men mutants). A majority of Gram-negative organisms utilize ubiquinone (Q) under aerobic conditions, and menaquinone under anaerobic conditions in their electron transport systems. Most importantly, the electron transport chain in humans does not utilize menaquinone. Clearly, the electron transport chain is a central component in the production of ATP and the subsequent growth of bacteria (Figure 3). Therefore, inhibitors of menaquinone biosynthesis or specific inhibitors of enzymes associated with electron transport systems have great potential for the development of novel and selective drugs against multi-drug resistant (MDR) Gram-positive pathogens. As mentioned above, the role of vitamin K_1_ in blood coagulation cascade, related biological pathways, and the function of vitamin K_2_ in bacterial electron transport have been studied independently. Although the functions of vitamin K_1_ in humans and vitamin K_2_ in bacteria are completely different, drug discovery targeting vitamin K_2_ or its biosynthesis requires careful consideration of vitamin K distribution in tissue (because of structural similarity of both vitamin Ks) and selectivity against the target protein (because essential vitamin K-dependent protein(s) may be interfered by vitamin K mimics or vitamin K biosynthesis inhibitors). 

## 6. Biosynthesis of Menaquinone

Menaquinones are constituents of bacterial cytoplasmic membranes, and play an important role in electron transport, oxidative phosphorylation, active transport, and endospore formation in some Gram-positive bacteria [37]. The biosynthetic steps leading to menaquinone have been studied extensively in *E. coli* [38,39,40,41,42,43]. In *E. coli* the synthesis of menaquinone is accomplished by seven enzymes (MenA-MenG). These enzymes are encoded by 2 clusters of genes. The *men* gene cluster consists of the *menB*, *C*, *D*, *E*, *F* and a separate cluster containing *menA* and *menG* [41,44].

**Figure 4:**
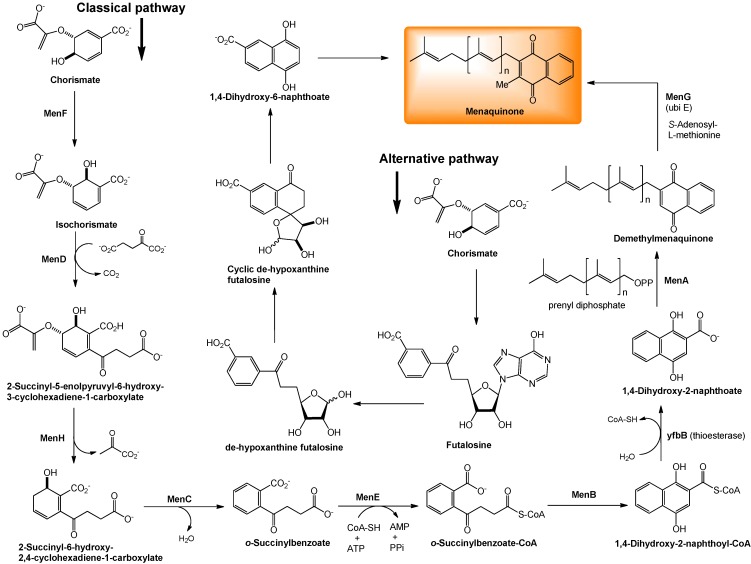
Biosynthesis of menaquinone.

The biosynthesis of menaquinone is initiated from chorismate (an intermediate for biosynthesis of aromatic amino acids, indole derivatives, tryptophan, salicylic acid, and many alkaloids) and proceed through a series of menaquinone-specific reactions. As summarized in Figure 4, MenF isomerizes chorismate to isochorismate, and MenD (a thiamine diphosphate-dependent enzyme) catalyzes a Stetter-like conjugate addition (a 1,4-addition of an carbonyl molecule to α,β-unsaturated compound) of α-ketoglutarate with isochorismate to form 2-succinyl-5-enolpyruvyl-6-hydroxy-3-cyclohexadiene-1-carboxylate, whose pyruvate moiety is eliminated by MenH to yield 2-succinyl-6-hydroxy-2,4-cyclo-hexadiene-1-carboxylate. MenC catalyzes aromatization of 2-succinyl-6-hydroxy-2,4-cyclohexadiene-1-carboxylate to form *o*-succinylbenzoate. MenE is an *o*-succinylbenzoate-CoA ligase that converts *o*-succinylbenzoate to *o*-succinylbenzoate-CoA. MenB subsequently catalyses a formal Dieckmann-type condensation of *o*-succinylbenzoate-CoA to afford 1,4-dihydroxy-2-naphthoyl-CoA, which is then hydrolysed to 1,4-dihydroxy-2-naphthoate (DHNA) by a thioeterase encoded by *yfbB*. On the other hand, the prenyl diphosphate of the appropriate size (n = 7 in *E. coli*, Figure 4) is biosynthesized by the chain-elongation reaction (iterative reaction of allyl diphosphate with isopentenyl diphosphate). DHNA is subsequently prenylated and methylated by MenA and MenG, respectively, resulting in the formation of menaquinone. Menaquinones are known to have side chains of different sizes in different organisms and sometimes even within the same organism. The most common menaquinones contain 7, 8 and 9 isoprene units; MK-7 is the major menaquinone component in many Gram-positive spore-forming bacteria, MK-8 is in *E. coli* (*vide supra*), and MK-9 in *M. tuberculosis*. 

However, menaquinones containing 4, 5, 6, 10, 11, 12, and 13 isoprene units have been reported in bacteria. Menaquinones are the predominant isoprenoid lipoquinones of Gram-positive bacteria, whereas Gram-negative bacteria such as *E. coli* and close relatives (*i.e*., enterobacteria) use menaquinone (MK), demethylmenaquinone (DMK), and ubiquinone (Q) in their electron transport chains (see Figure 1). Recent studies have shown that several representatives of the γ-proteobacteria appear to share the similar electron transport system to that observed in *E. coli* [45]. Several studies indicated that the regulation of menaquinone biosynthesis seems to be different from that of the enzymes of anaerobic respiration that are controlled by the general regulator FNR [46,47,48,49]. In addition, the MK/DMK ratio is independent of the *fur* locus and high levels of naphthoquinones (MK and DMK) are found only under anaerobic conditions in *E. coli*. Menaquinones were found almost exclusively (85~90%) in the bacterial membrane. *The total content of naphthoquinones was reported to be between 0.60~1.09 μmol/g cell protein* (present in a larger amount in *C. thermoaceticum*: 1.80 μmol/g cell protein). It is evident that several bacterial species do not have methylase (or MenG) and produce DMK as their sole quinone [47,50,51]. Conversion of DMK to MK is the last step in the biosynthesis (Figure 4). Most likely, the activity of the DMK methylase (*i.e*., MenG) is regulated by the presence or absence of the electron carriers or by the supply of *S*-adenosylmethionine (co-factor of MenG) [52]. 

A bioinformatic analysis of whole genome sequences has suggested that some microorganisms, including pathogenic species such as *Helicobacter pylori*, *Campylobacter jejuni*, and lactobacilli do not have orthologs of the *men* genes, although they synthesize menaquinone. Recent studies showed that these bacteria synthesize menaquinones in an alternative pathway *via* futalosine as illustrated in Figure 4 [45]. 

## 7. Targeting Electron Transport System in Drug Discovery

### 7.1. NADH Dehydrogenase

**Figure 5:**
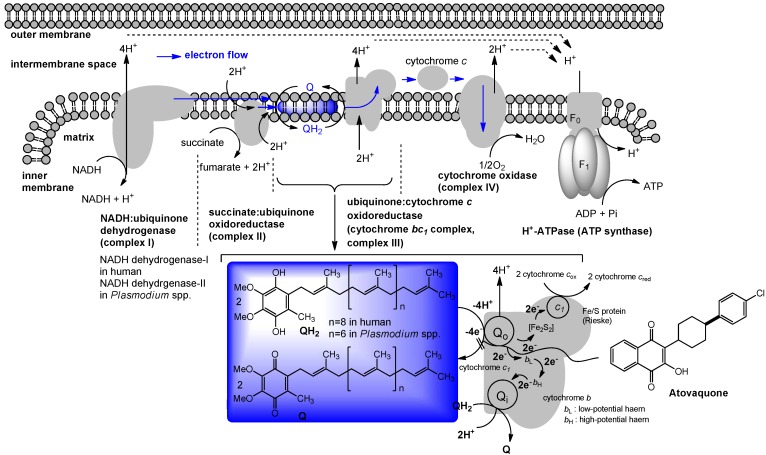
Schematic representation of mitochondrial electron transport chain and the proposed binding site of atovaquone.

Maintenance of mitochondrial membrane potential plays essential roles in cellular energy production. For example, the mitochondrial electron-transport chain is essential for survival in malaria and is a validated target for antimalarial drugs. The role of *P. falciparum* type II NADH:quinone oxidoreductase (PfNDH_2_) in the mitochondrial electron-transport chain has been reviewed [53,54,55]. There are at least five mitochondrial dehydrogenases, including PfNDH_2_, which actively oxidize NADH in the presence of ubiquinone (Q). In these processes, Q is reduced to form hydroquinone (QH_2_) and transfer two electrons to ubiquinone:cytochrome *c* oxidoreductase (complex III) in which QH_2_ is in turn reoxidized to Q similar to the bacterial electron transport system summarized in Figure 3. The molecular mechanism of the inhibitors of the quinol oxidase (Q_o_) site of complex III was characterized by X-ray crystallography. The binding mode of atovaquone, a drug used to treat malaria and *Pneumocystis carini*, to the yeast complex III was analyzed by the enzyme kinetic studies (*Ki* = 9 nM). The differential inhibition of the fungal versus mammalian enzymes was also described [56]. A schematic respiration of the mitochondrial electron transport chain and the proposed binding site of atovaquone are illustrated in Figure 5. 

Although a successful drug development targeting the electron transport system of malaria parasite is known (*vide supra*), there are a few studies that investigated the electron transport system for development of new antibacterial drugs. Recently, Weinstein and co-workers reported inhibitors of type II NADH:menaquinone oxidoreductase that effectively killed *M. tuberculosis in vitro* [57]. Human mitochondria use only type I NADH dehydrogenase, whereas *M. tuberculosis* possesses both type I and II NADH dehydrogenases. Interestingly, the level of transcription of type I NADH dehydrogenase is down-regulated upon starvation *in vitro* and type I NADH dehydrogenase seems to be dispensable for growth of *M. tuberculosis in vitro*, classic inhibitors of type I NADH dehydrogenase, such as rotenone, piericidin, and pyridaben (see Figure 7), did not inhibit *M. tuberculosis* NADH hydrogenase activities. The same authors observed that addition of NADH to *M. tuberculosis* membrane fractions resulted in a linear consumption of O_2_, but that consumption was inhibited by the addition of a series of phenothiazine molecules. Respiration of the drug-arrested membranes was restored by the addition of a mixture of ascorbate and tetramethyl-*p*-phenylenediamine which donate electrons at the level of cytochrome *c* [57].

**Figure 6:**
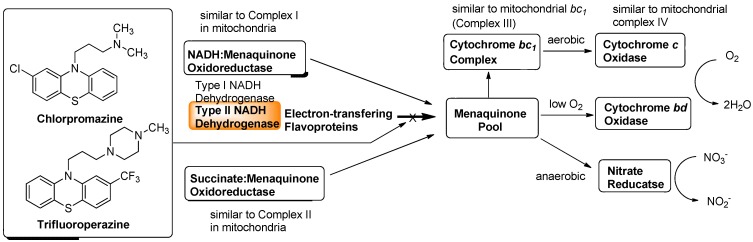
Electron flow in *M. tuberculosis* and type II NADH dehydrogenase inhibitors.

This observation implied that phenothiazines target upstream of cytochrome *c*. Enzymatic inhibitory assays of the phenothiazine molecule against a series of oxidases (cytochrome *bd* oxidase) and oxidoreductases [the cytochrome *bc_1_* complex (ubiquinol:ferricytochrome *c* oxidoreductase), type II NADH dehydrogenase, and succinate dehydrogenase (complex II)] were performed, and it was observed that phenothiazines inhibited only type II NADH dehydrogenase. Thus, type II NADH dehydrogenase could be a unique and interesting antimicrobial target [57,58,59] (Figure 6).

### 7.2. Other Respiration Inhibitors

**Figure 7:**
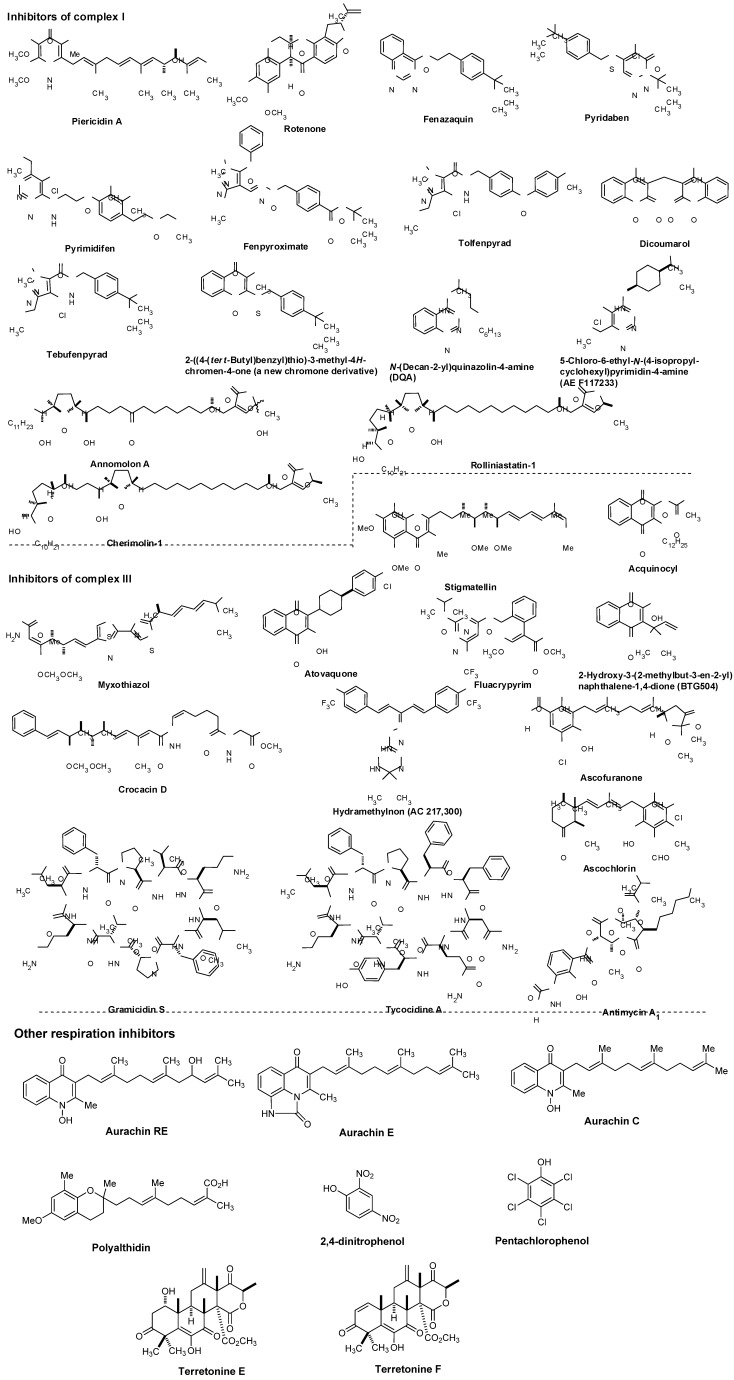
Representative known respiration inhibitors.

Representative respiration inhibitors which exhibited antiviral, antibacterial, or antifungal activity are summarized in Figure 7. The molecules interfering with the electron transport chain in mitochondria are also included in Figure 7. Some of the listed molecules do not selectively inhibit target respiratory enzyme(s), and thus, cytotoxicity of such molecules was also reported. However, mitochondrial respiration inhibitors having relatively low mammalian toxicity and short environmental persistence have been developed for use as insecticides and acaricides (*i.e.*, pyridazine, pyrazole, quinazoline, naphthoquinone, pyrroles, and pyrimidines as summarized in Figure 7) [60]. Moreover, each molecule listed in Figure 7 is very important as an investigative tool in cell biology. 

NADH:ubiquinone oxidoreductase (complex I, Figure 5) is reported to drive about 2/3 of the ATP synthesis by oxidative phosphorylation [61]. Classic complex I inhibitors are rotenone and piericin A. Rotenone inhibits the transfer of electrons from iron-sulfur centers in complex I to ubiquinone and has been utilized as a broad-spectrum insecticide, piscicide, and pesticide [62]. Pyridaben, pyrimidifen, and fenpyroximate are inhibitors of mitochondrial electron transport at complex I, and have been utilized as pesticides, insecticides, or herbicides [63]. Tebufenpyrad, tolfenpyrad and fenazaquin are pyrazole acaricides and insecticides. Fenazaquin inhibits electron transport chain by binding with complex I at the Q_o_ site and shows excellent performance against pests that are resistant to existing insecticides [64]. Tolfenpyrad is a broad spectrum insecticide and has not exhibited cross resistance with current products which were developed for use in vegetables, in particular cruciferous leafy vegetables, fruits and other high-value markets. Dicoumarol is an anticoagulant which functions as a vitamin K antagonist (similar to warfarin), and is also a competitive inhibitor of NADH:quinone oxidoreductase 1 (NDH2), increasing intracellular superoxide and affects cell growth of tumor cells [65]. 2-Benzyl-mercaptochromones were proposed as mimics of quinone or hydroquinone. A chromone derivative (Figure 7) showed a selective inhibitor of complex I and acaricidal activity against spider mites [66]. Several aminopyrimidine molecules such as DQA and AE F117233 were developed for selective inhibitors of the quinone-binding sites in mitochondrial NADH:ubiquinone oxidoreductase [67]. Annonaceous acetogenins (ACGs) are a group of secondary metabolites isolated from the family Annonaceae, many of which exhibit high cytotoxic and antitumour activities [68,69]. The structures of representative molecules, rolliniastatin-1, annomolon A, and cherimolin-1, in this family were shown in Figure 7. The biological effect of these substances are attributed to the inhibition of mammalian mitochondrial NADH-ubiquinone oxidoreductase and to the inhibition of a ubiquinone-linked NADH oxidase expressed in the plasma membrane of cancerous cells.

The cytochrome *bc_1_* complex (also known as ubiquinol-cytochrome *c* reductase or complex III, Figure 5) is the central segment of the energy-conserving, electron transfer chain of mitochondria and many respiratory and photosynthetic bacteria. This enzyme complex catalyzes electron transfer from ubiquinol to cytochrome *c* with concomitant translocation of protons across the membrane to generate a proton electrochemical gradient required for ATP synthesis by ATP synthase [70]. 

As illustrated in Figure 5, the cytochrome *bc_1_* complexes contain three core subunits, cytochrome *b*, cytochrome *c_1_*, and Rieske iron-sulfur protein (ISP), however, the cytochrome *c*_1_ subunit is not always part of the bacterial cytochrome *bc_1_* complex. The three-dimensional crystal structures of mitochondrial *bc_1_* complexes from bovine, chicken, and yeast have been elucidated [71,72,73,74,75,76,77]. In general, high-affinity inhibitors of the cytochrome *bc_1_* complex target either the quinol oxidation site (Q_o_), or the quinone reduction site (Q_i_). The antimycins were discovered as potent fungicides. Antimycin A_1_ is one of the most predominant forms and works specifically by targeting the Q_i_ site of the cytochrome *bc_1_* complex in most respiratory and photosynthetic organisms [78,79]. Because of specific binding to complex III, antimycin A_1_ has been found to be toxic to all organisms which are dependent on mitochondrial respiration for their energy source. Myxothiazol blocks electron transport between quinol and the ISP [80], and inhibits the growth of many yeasts and fungi, but is generally inactive or very weak against bacteria. Crocacin D inhibits the growth of Gram-positive bacteria and a wide spectrum of yeasts and mold through blocking the electron transport within the cytochrome *bc_1_* [81]. Stigmatellin, a quinol oxidation site (Q_o_) inhibitor, blocks electron transfer from iron-sulfur protein (ISP) to cytochrome *c_1_* in the *bc_1_* complex [82]. The binding site and mode of action of the antimalarial drug, atovaquone, was described in **7.1**. Fluacrypyrim is an acaricide, and specifically inhibit the ubiquinol oxidizing site Q_o_ of the cytochrome *bc_1_* [83]. Acequinocyl is a proacaricide that forms the deacylated active metabolite, and the active form inhibits the respiration of mitochondria at complex III in the electron transport system [84,85]. Similarly, a 3-hydroxynaphthoquinone, BTG505, is an active hydroquinone insecticide that is utilized for a pro-insecticide approach. Hydramethylnon (know as AC 217,300) is used primarily as an insecticide in the form of baits for cockroaches and ants, and its mode of action was described as an inhibitor of complex III [86]. Ascofuranone and ascochlorin are structurally related to ubiquinol and specific inhibitors of the cytochrome *bc_1_* complex. Ascochlorin showed significantly more active in inhibiting complex III than ascofuranone [87,88]. Ascochlorin was also reported to inhibit matrix metalloproteinase-9 (MMP-9) in human renal carcinoma (Caki-1) cells [89]. Natural peptide antibiotics such as the gramicidins (a heterogeneous mixture of six antibiotic compounds) have been used extensively for topical therapy with excellent results. Mechanistically, gramicidin S and tyrocidine A associate with bacterial cell membranes and cause inhibition of cytochrome *bd*-type quinol oxidase (a prokaryotic terminal oxidase catalyzing O_2_ reduction to H_2_O), whereas succinate oxidase and cytochrome *c* oxidase activities are considerably less sensitive to these molecules [90]. 

Pentachlorophenol and 2,4-dinitrophenol are biocidal agents, and have been known to inhibit enzymes involved in energy metabolism. The aurachins (structures of aurachin C, E, and ED shown) showed inhibitory activities against Gram-positive bacteria and a few yeasts and molds by blocking NADH oxidation [91,92,93,94]. Polyalthidin was reported to inhibit the mitochondrial electron transport chain complex [95]. Terretonins E and F were isolated from marine-derived fungus, *Aspergillus insuetus*. These molecules displayed activity as inhibitors of the mammalian mitochondrial respiratory chain [96].

As described above, NADH hydrogenase is an established drug target for the malaria parasite and is likely to be an attractive target for *M. tuberculosis* infections. Although these electron transport chain complexes and their inhibitor molecules discussed in chapters 7.1 and 7.2 are not directly related to vitamin K, validated targets in the electron transport systems and FDA approved drugs targeting this system encourage us to discover novel drugs which selectively target the electron transport system of pathogens (*vide infra*). In addition, it will be considerably important to investigate an effective combination of electron transport inhibitor (*i.e*., menaquinone biosynthesis inhibitor) and electron transport chain complex inhibitor (*i.e*., NADH dehydrogenase) in the development of novel antibacterial drugs targeting bacterial respiratory system.

## 8. Menaquinone Biosynthesis as a Target for Antibacterial Agents

**Figure 8:**
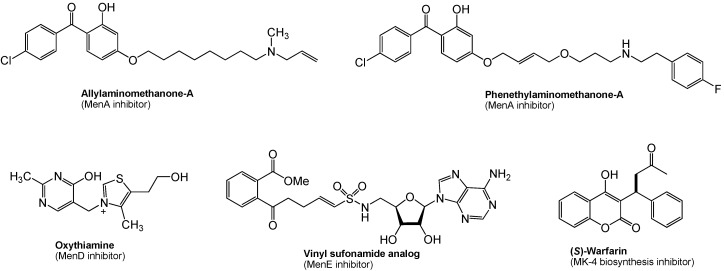
Representative menaquinone biosynthesis inhibitors.

As described in Section 4, menaquinone is the sole quinone in the electron transport chain in the majority of Gram-positive bacteria. Since the pathway leading to the biosynthesis of menaquinone is absent in humans, the bacterial enzymes catalyzing the synthesis of menaquinone are potential novel targets for novel antibacterial drug discovery. In *M. tuberculosis*, it is speculated that dormant (nonreplicating) bacilli are considered to have a less active metabolism and less energy reserves, however, ATP synthesis in oxidative phosphorylation must be active even in the dormant form. Thus, inhibition of menaquinone synthesis could have profound effects on maintenance of dormancy in *M. tuberculosis*. This concept is supported by several reports that phenothiazines inhibited Type II NADH:menaquinone oxidoreductase (Figure 6), the first enzyme in bacterial respiratory chain, and were effective in killing nonreplicating *M. tuberculosis* [57]. It was recently demonstrated that (1) inhibition of MenA (1,4-dihydroxy-2-naphthoate prenyltransferase) (Figure 4) showed significant growth inhibitory activities against drug-resistant *Mycobacterium* spp. [97], and (2) MenA inhibitors were effectively killed dormant *M. tuberculosis in vitro* using the Wayne model [98,99,100]. Several other promising biological date were generated on MenA as a new drug target; (1) *M. tuberculosis* growth *in vitro* could not completely be rescued by supplementation with ~400 μM of a vitamin K_2_, and (2) all Gram-positive bacteria tested were susceptible to MenA inhibitors, whereas Gram-negative bacteria were not susceptible (either do not have the target or the target is only expressed in anaerobic conditions) [101,102]. Reported representative menaquinone biosynthesis inhibitors are summarized in Figure 8. Significantly, allylaminomethanone-A exhibited a 320-, 180- and 3-fold more effective in killing nonreplicating bacteria *in vitro* than ethambutol, isoniazid or rifampicin (1^st^ line TB drugs) at the concentrations of 10 μg/mL; allylaminomethanone-A exhibited the most active in killing nonreplicating *M. tubeculosis in vitro* among antimycobacterial agent tested [100]. In addition, MenA inhibitors (Figure 8) inhibited growth of drug resistant *Mycobacterium* spp. and other Gram-positive bacteria at low concentrations [97]. Thus, these observations are expected to be of significance in discovering new lead molecules to combat Gram-positive pathogens which biosynthesize menaquinone through “classical pathway” as summarized in Figure 4. 

In menaquinone biosynthesis (Figure 4), MenD (2-succinyl-5-enoylpyruvyl-6-hydroxy-3-cyclohexane-1-carboxylic acid synthase), MenE (an acyl-CoA synthase), and MenB (1,4-dihydroxynaphtoyl-CoA synthase) have recently been studied for the development of novel drug lead for Gram-positive pathogens including *M. tuberculosis* [103,104,105,106]. Oxythiamine derivatives were found to be antibacterial MenD inhibitors. A series of vinyl sulfonamides were designed based on MenE enzymatic substrate and an adenylated molecule showed an excellent MenE enzymatic inhibitory activity. Although structures have not yet been in the public domain, a series of MenB inhibitors were discovered *via* a high-throughput screen against MenB. (*S*)-Warfarin, vitamin K epoxide reductase inhibitor (see, Figure 1), was reported to inhibit the biosynthesis of MK-4 from menadione (Figure 2) in chick liver. Interestingly, (*R*)-warfarin showed approximately four times less active than its enantiomer in MK-4 biosynthesis inhibition [107]. 

## 9. Summary

The electron transport system couples with ATP synthase to produce ATP through oxidative phosphorylation. Bacterial ATP synthase, F_1_F_0_-ATPase, is a viable target for treatment of multi-drug resistant (MDR) *M. tuberculosis* infections. Diarylquinolone R208910 (TMC207, a Phase II clinical drug) as an inhibitor of ATP synthase exhibited a remarkable activity against mycobacteria [108,109]. As discussed in **7.1**, Type II NADH hydrogenase is a promising target for *M. tuberculosis* infections. On the other hand, menaquinone biosynthesis has been neglected as targets for the development of novel antibacterial agents. Menaquinone is a key component of the electron transport systems in the majority of Gram-positive bacteria including *M. tuberculosis*. Recently, inhibitors of menaquinone biosynthesis in Gram-positive bacteria have been identified (Figure 8), and these compounds are also effective inhibitors of bacterial growth. In development of new drugs for *M. tuberculosis* infections, it is the ultimate goal to discover an antimycobacterial drug which is effective against human latent tuberculosis infection. It has not been determined whether menaquinone biosynthesis enzyme genes are expressed in any growth conditions in *M. tuberculosis*. However, the mycobactericidal effect of the representative MenA inhibitor against nonreplicating *M. tuberculosis* strongly suggest that electron transport is important in maintaining bacterial viability under conditions of restricted oxygen. Several data suggest a role for the DosR/DosS/DosT signaling system, which is required for *M. tuberculosis* genetic response to hypoxia and nitric oxide, in the adaptation of *M. tuberculosis* to conditions that trigger reversible bacterial stasis *in vitro*, and thus, the DosR/DosS/DosT signaling system may contribute to latency *in vivo* [110]. MenA inhibitors are able to block the electron flow without inducing a dormancy response in *M. tuberculosis*. It is conceptually very interesting that menaquinone biosynthesis inhibitors can be developed as indirect ATP synthesis inhibitors. 

To date, no data is available regarding vitamin K uptake in bacteria when bacteria are deficient in menaquinone. *In vitro* rescue studies indicate that *M. tuberculosis* treated with a MenA inhibitor could not be rescued completely even at 400 μM concentrations of exogenous vitamin K_2_ (*vide supra*), and thus, menaquinone deficient bacteria is not likely to attain the normal levels of electron transport components (total vitamin K concentrations is 0.47~1.19 nmol/L in plasma and is significantly low in tissues) through passive transport (or facilitated diffusion). In addition, several studies indicated that menaquinone-deficient mutants of some Gram-positive bacteria were characterized as reduced growth and deficiencies in cytochromes *a*, *b*, and *c* (in *B. subtilis*) [111,112]. Many biological functions are associated with the maximal amounts of menaquinone in membranes. Nonetheless, it is important to prove the efficacy of menaquinone biosynthesis inhibitor using an appropriate infected animal model; a menaquinone biosynthesis inhibitor has to demonstrate efficient eradication of infected bacteria *in vivo*. Thus, discovery of a pharmacologically acceptable menaquinone biosynthesis inhibitor which possesses a significant antibacterial activity should be emphasized for a pharmacological proof-of-concept of menaquinone biosynthesis as drug targets. In addition, combination therapy will remain mandatory to combat MDR-pathogens including *M. tuberculosis*. In this respect, ATP synthase inhibitors and/or other respiratory inhibitors (*i.e*., NADH hydrogenase inhibitor) are expected to show an enhanced synergy effect with menaquinone biosynthesis inhibitors. 
